# Systematic scoping review of the implementation, adoption, use, and effectiveness of digital contact tracing interventions for COVID-19 in the Western Pacific Region

**DOI:** 10.1016/j.lanwpc.2022.100647

**Published:** 2023-02-25

**Authors:** Melanie Bannister-Tyrrell, Mengji Chen, Vladimir Choi, Alessandro Miglietta, Gauden Galea

**Affiliations:** a Nossal Institute for Global Health, 333 Exhibition Street, Melbourne, Victoria, 3004, Australia; b World Health Organization Regional Office for the Western Pacific, Manila, Philippines; c World Health Organization Representative Office in China, Beijing, China

**Keywords:** Digital health, COVID-19, Contact tracing, Digital contact tracing, Health emergencies

## Abstract

A systematic scoping review of digital contact tracing (DCT) interventions for COVID-19 was conducted to describe the implementation, adoption, use and effectiveness of DCT interventions implemented as part of the COVID-19 response in the Western Pacific Region (WPR). A systematic search identified 341 studies and 128 grey literature sources, of which 18 studies and 41 grey literature sources were included. 17 (46%) WPR countries and areas implemented DCT interventions. Adoption ranged from 14.6% to 92.7% in different adult populations and epidemiological contexts. Trust in authorities, and privacy concerns and beliefs, were the most frequent determinants of adoption and use. Only two studies analysed DCT effectiveness, which showed limited to no effectiveness of DCT interventions in low transmission settings. Overall, there is limited evidence available to evaluate the contribution of DCT to mitigating COVID-19 in the WPR. Preparedness for future health emergencies should include developing robust frameworks for DCT effectiveness evaluations.

**Research in context**

**Evidence before this study**

In June 2020, the World Health Organization Regional Office for the Western Pacific published interim guidance to support countries and areas in the Western Pacific Region (WPR) to select, design, and implement digital contact tracing interventions (DCT) to reduce the spread of COVID-19. However, at the time this guidance was issued, there was limited empirical evidence for the public health effectiveness of DCT interventions for COVID-19 or other infectious diseases under real-world settings, with most supporting evidence arising from modelling studies. Additionally, modelling studies made assumptions about the adoption and use of DCT interventions that could not be empirically verified until after roll-out.

A search of peer-reviewed and preprint literature in PubMed and EMBASE using search terms including “COVID-19”, “digital contact tracing”, “exposure notification”, “proximity tracing”, “contact tracing app”, “use”, “acceptance”, “adoption”, “performance”, “effectiveness” and “impact” from 1st January 2020 to 4th April 2022 identified 255 unique records, including 12 articles describing a protocol or findings of a systematic, scoping, or rapid review. Of these, one study reviewed the public health effectiveness of DCT interventions globally, with a search cut-off of April 2021. This study did not describe the implementation characteristics of DCT interventions, including different DCT intervention designs and COVID-19 transmission contexts, and did not consider barriers or enablers of adoption or use.

**Added value of this study**

This study presents a comprehensive overview of the use of DCT interventions for COVID-19 in the WPR, including reviewing the implementation of DCT interventions in settings with limited or no detected transmission and COVID-zero policies in place for some time periods, as well as settings with widespread community transmission of COVID-19. Most of the available research focused on determinants of adoption or use of DCT interventions, and reported similar findings to global studies. This review highlights the evidence gap regarding the effectiveness of DCT interventions for COVID-19 in the WPR. Most studies estimated adoption or use at a single time point, in non-representative populations, and did not stratify findings by important social determinants of health, including access to digital health services. There were only two effectiveness studies of Bluetooth-based DCT smartphone apps in the WPR: a pilot validation study in Singapore, and a retrospective effectiveness evaluation in Australia. No study evaluated the contribution of Quick Response (QR)-code apps to reducing transmission, despite this being the most commonly used technology.

**Implications of all the available evidence**

Despite the considerable investment in and population-level use of DCT interventions throughout the WPR, there is limited high quality evidence available to evaluate the contribution of DCT interventions to mitigating COVID-19 transmission. This review also highlights that most available evidence about the adoption, use or effectiveness of DCT interventions derives from the first 12 months of the pandemic, prior to the emergence of several more highly-transmissible variants. This represents an important evidence gap, as the relevance of the available evidence on adoption, use and effectiveness of DCT interventions from the first year of the COVID-19 pandemic may not be generalisable to time periods dominated by more transmissible variants. An important element of future pandemic preparedness is for countries and areas to develop or adopt robust evaluation frameworks for DCT interventions prior to any future deployment, including ensuring the need for data availability for research and evaluation is balanced against privacy concerns.

## Introduction

Contact tracing is a fundamental public health intervention and a key component of the COVID-19 pandemic response. Digital contact tracing (DCT) interventions for COVID-19 were rapidly developed and implemented worldwide, following modelling in early 2020 suggesting that DCT interventions, if widely adopted, had the potential to contain transmission and avoid the requirement for mass quarantine and movement restrictions.^1^ Most COVID-19 DCT interventions intended for use by the general public have been deployed as smartphone applications (apps), though tokens and wearables have also been used in some settings.^2^ Even modest levels of adoption and use of DCT apps were estimated to contribute to reducing COVID-19 transmission, if implemented alongside other public health and social measures.^1^^,^^3^

Globally, evidence for the effectiveness of DCT interventions for COVID-19 from empirical studies is mixed, and generally DCT interventions have performed poorer in real-life settings than estimated from modelling studies. A systematic review of empirical effectiveness studies published in English up to April 2021 identified 10 studies worldwide that reported measures of DCT adoption and use, of which one study reported the ratio of exposure notifications received to positive test results, and none reported on app performance in preventing further transmission.^4^ At the time of this prior review, no study reported the effectiveness of DCT apps compared to conventional contact tracing,^4^ though this has since been assessed in one study from Australia, which demonstrated no increased effectiveness of DCT compared to conventional contact tracing in a high-resource low-transmission setting.^5^ However, comparisons of DCT effectiveness compared to conventional contact tracing are highly dependent on the epidemiological context.^6^ Another study published since this prior review estimated that in the United Kingdom, during a period with high incidence of COVID-19 and with limited to no conventional contact tracing occurring, each COVID-19 case using a DCT app contributed to one COVID-19 case averted, though with considerable uncertainty about the absolute number of COVID-19 cases averted during the study period (108,000–914,000 across two different estimation methods).^7^

In June 2020, the World Health Organization Regional Office for the Western Pacific (WPRO) published interim guidance to support countries and areas in the Western Pacific region (WPR) to select, design and implement DCT interventions to reduce the spread of COVID-19. At the time this guidance was issued, there was limited empirical evidence for the effectiveness of DCT interventions for COVID-19 or other infectious diseases under real-world settings. Many countries and areas in the WPR introduced DCT interventions to support their COVID-19 response, utilizing different technologies, functionalities and implementation models. More than two years since the rollout of DCT interventions in the WPR, the overall impact of DCT interventions in the WPR has not been assessed. Lessons learned about the implementation, adoption, use, and effectiveness of DCT interventions assessed in studies and systematic reviews of DCT interventions, which predominantly include studies in high-income European or North American settings, may not fully reflect the WPR given its globally unique COVID-19 epidemiological context, as well as geographical and geopolitical diversity. The incidence of COVID-19 in the WPR was the lowest of all World Health Organization (WHO) regions until the end of 2021, and several countries and areas in the region reported zero community-acquired COVID-19 cases nationally or in subnational jurisdictions for several months at a time, repeatedly controlling local outbreaks and eliminating local transmission.^8^ This early success in controlling COVID-19 in WPR countries and areas may be attributable to several factors, including prior development of and investment in preparedness and response plans, closed international borders, COVID-zero policies, prolonged lockdowns, compliance with public health and social measures, and other factors.^9^^,^^10^ Reduced testing requirements in some regions in 2022 makes direct comparisons of COVID-19 incidence between them challenging. However, as of June 2022, reported deaths due to COVID-19 in the WPR were the second-lowest of all regions, after the African region.^11^

Conducted as part of an operational review of the COVID-19 response in the WPR, the present study aims to document experiences and review lessons learned from the use of DCT interventions, to strengthen national and regional preparedness for future health emergencies in the WPR. Focusing on government-endorsed DCT interventions rolled out country- or area-wide, and designed for use by the general population, this review aims firstly to describe the implementation of DCT interventions to support the COVID-19 response in the WPR, and secondly to analyse the adoption, use, and effectiveness of implemented interventions, which are key for interpreting their public health impact.

## Methods

### Overview

This study was designed as a systematic scoping review following the Preferred Items for Systematic Reviews and Meta-Analysis guidelines extension for Scoping Reviews (PRISMA-ScR).^12^ A systematic scoping review was conducted of DCT interventions deployed as part of the COVID-19 pandemic response in countries and areas in the WPR, a region encompassing more than one quarter of the global population. The types of tools that comprise DCT interventions include smartphone applications, physical tokens, or wearables, and the types of technologies that support contact tracing include Bluetooth proximity tracing, Quick Response (QR) code location check-in, GPS tracking, radio frequency signals and other approaches.^2^ In addition to describing the characteristics of the implementation of DCT interventions, the public health outcomes of interest were the adoption, use and effectiveness of DCT interventions. As this research was initiated as part of the operational response to the COVID-19 pandemic, a review protocol was not registered, though a detailed terms of reference document for the review was prepared for internal use.

### Eligibility criteria

Studies, grey literature, and other information sources were eligible for inclusion in this review if the source reported empirical data on the implementation, adoption, use (including barriers and enablers of adoption or use), and/or effectiveness measures of a COVID-19 DCT intervention that was designed for use by the general population, and which formed part of a national government's COVID-19 response in any of the 37 countries and areas that comprise the WHO WPR. Modelling studies based on simulated data were excluded. DCT interventions designed for use exclusively by public health professionals, such as software designed to support contact tracing data management, visualisation, and interpretation, were excluded. Sub-national DCT interventions, and private sector DCT interventions that were not endorsed as part of a government COVID-19 response, were excluded.

The countries and areas in the WHO WPR are American Samoa, Australia, Brunei Darussalam, Cambodia, China, Cook Islands, Fiji, French Polynesia (France), Guam (USA), Hong Kong Special Administrative Region (SAR) (China), Japan, Kiribati, Lao People's Democratic Republic (Lao PDR), Macao SAR (China), Malaysia, Marshall Islands, Federated States of Micronesia, Mongolia, Nauru, New Caledonia (France), New Zealand, Niue, Commonwealth of the Northern Mariana Islands (USA), Palau, Papua New Guinea, Philippines, Pitcairn Island (UK), Republic of Korea, Samoa, Singapore, Solomon Islands, Tokelau (New Zealand), Tonga, Tuvalu, Vanuatu, Viet Nam, and Wallis and Futuna (France).

### Information sources

Information sources for this scoping review comprised peer-reviewed journal articles, preprint articles, grey literature, and other information sources. Two separate search strategies were used. Firstly, as substantial information about DCT implementation was expected to be available in grey literature and other information sources, an open-ended search was performed. Google web search was used to identify studies, technical reports or guidance from governments or international health agencies, government press releases and online news media describing any aspect of DCT implementation in WPR countries and areas. Secondly, a systematic search of the peer-reviewed and preprint literature published between 1st January 2020 and 4th April 2022 using PubMed and EMBASE (with search in EMBASE inclusive of preprint studies published on the medRxiv and bioRXiv servers), without language restrictions, was conducted to identify studies and information sources reporting on the public health outcomes of interest; namely adoption, use, and effectiveness.

### Search

The search for studies reporting on adoption, use, and effectiveness outcomes was performed in PubMed and EMBASE, using the following search terms (shown as constructed in PubMed):

“COVID-19” AND (“digital contact tracing” OR “exposure notification” OR “proximity tracing” OR “proximity tracking” OR “contact tracing app”) AND (“use” OR “acceptance” OR “adoption” OR “performance” OR “effectiveness” OR “impact”)

### Selection of sources of evidence

One reviewer (MBT) screened titles and abstracts retrieved through the database search to identify potentially relevant studies, and then assessed full text articles for eligibility for inclusion as part of the systematic scoping review to evaluate adoption, use, and effectiveness outcomes using COVIDENCE software. Four reviewers (MBT, VC, AM, MC) conducted the open-ended web search for descriptive information on DCT implementation and imported records into a Zotero library.

### Data charting process

Data from studies retrieved through the systematic search were extracted using COVIDENCE using pre-defined and piloted forms, and exported to Microsoft Excel for analysis. Data on DCT implementation from grey literature and other information sources were extracted into a Microsoft Excel spreadsheet for management and further processing.

### Data items

Data items collected for all information sources included name and country (ies) or area(s) of DCT implementation, technology(ies) used, and other implementation characteristics (e.g., launch date, mandatory or voluntary use). For information sources reporting on the public health outcomes of interest, additional data items including study authors, data collection period, and study population characteristics were collected, as well as data on the outcomes of interest, and data reported for the other three outcomes (adoption, use and effectiveness). The outcome definitions were aligned with the WHO/ECDC indicator framework for the public health effectiveness of digital proximity tracing solutions, with adaptation to account for the full range of DCT tools in use (e.g. non-app-based tools, QR code check-in apps, etc), and to allow inclusion of qualitative or categorical data as well as quantitative data. Specifically, ‘adoption’ was defined as proportion of the population that downloaded’ a DCT app, or receipt of tokens or wearable technologies. ‘Use’ was defined as active or regular engagement with the DCT intervention, such as conducting location check-ins using QR code-based apps, having proximity tracing apps open and/or with Bluetooth enabled as required, or any reports of frequency of use (e.g., daily, weekly). Qualitative or quantitative data on factors associated with higher use (i.e. enablers) or lower use (i.e. barriers) was also collected, regardless of whether quantitative data on frequency of adoption or use was also presented. Whether adoption or use measures were reported in different subpopulations (e.g. confirmed COVID-19 cases, or higher risk groups) was captured as a binary variable, and all adoption and use measures were narratively summarised. Quantitative and qualitative data reporting on factors associated with adoption or use were categorised as ‘positively associated with adoption/use’, ‘negatively associated with adoption/use’, or ‘not associated with adoption/use’. Factors associated with adoption or use were qualitatively thematically analysed and categorised as ‘privacy concerns and beliefs’, ‘trust in authorities’, ‘benefits to individuals’, ‘benefits to community’, ‘community attachment’ amongst others. In this emerging field of research, there are many different possible measures of effectiveness, some of which have been previously defined in the WHO/ECDC indicator framework for the effectiveness of digital proximity tracing.^13^ Effectiveness outcomes included all measures of the frequency or timeliness with which DCT interventions detected contacts at risk of infection, including estimates of the frequency or timeliness of detection of contacts who were confirmed cases compared to contacts who did not test positive. As this indicator framework was published after the start of the eligibility period for inclusion of studies in this review, all effectiveness measures that met the broad criteria defined above were extracted as reported in the information source and narratively summarised.

### Synthesis of results

Studies, grey literature, and information sources were grouped by country/area, and data on implementation, adoption, and use were tabulated and narratively summarised. Data on effectiveness measures were summarised in the text. Quantitative meta-analysis was not performed, as studies reported a range of different measures, often in non-representative populations. Where multiple studies provided outcome estimates for the same DCT intervention, the range was reported. Categorised factors associated with adoption or use were summarised for individual studies, and added across studies.

## Results

### Selection of sources of evidence

**Fig. 1:**
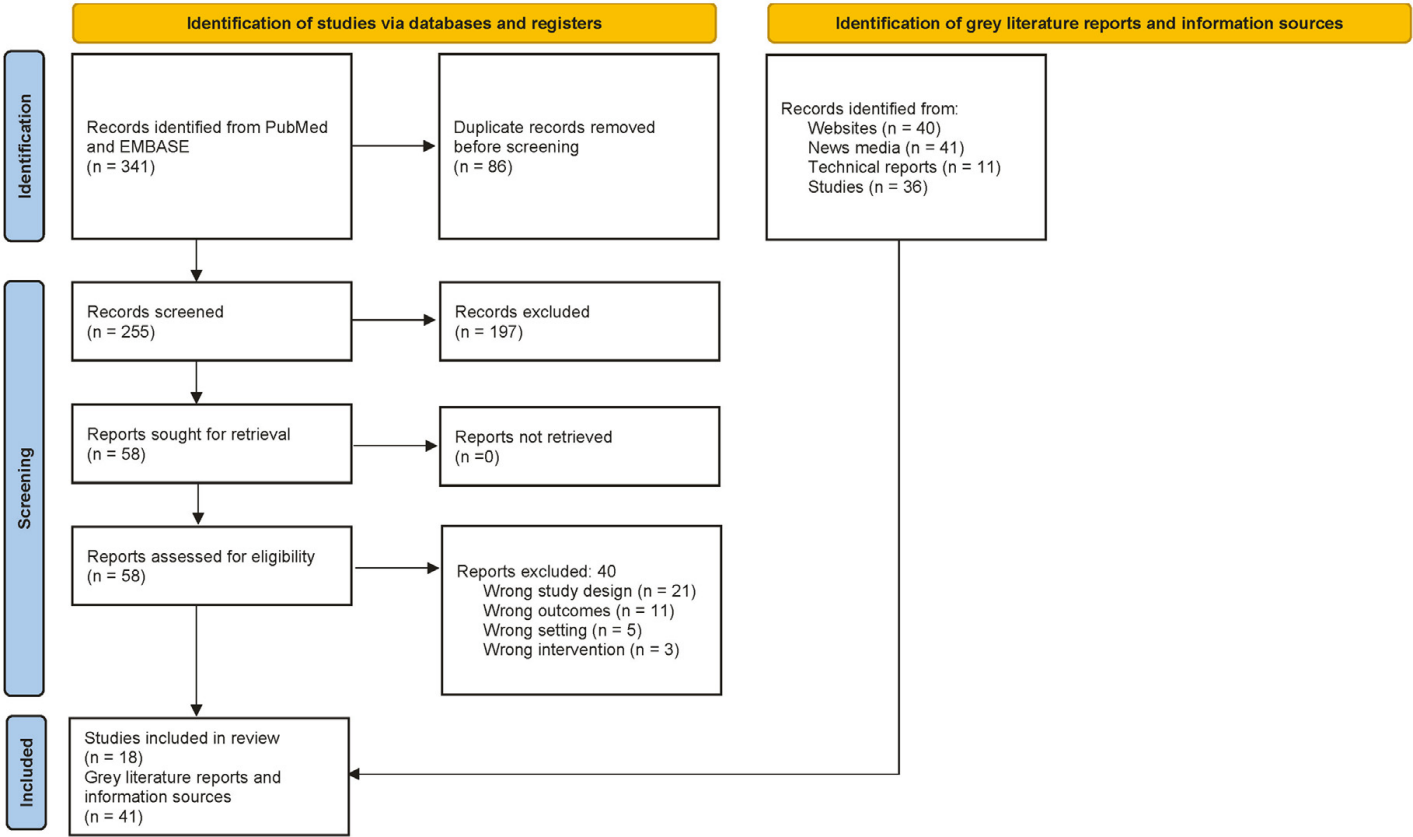
Selection of sources of evidence.

For the systematic search of adoption, use, and effectiveness of DCT interventions in the WPR, 341 references were retrieved from the database searches, of which 255 studies were screened and 58 studies assessed for full-text eligibility. After excluding 21 studies with an ineligible study design (review articles (n = 12), modelling studies (n = 5) and other study types (n = 4)), 11 studies reporting no measures of adoption, use, or effectiveness, five studies conducted only in sub-national settings focusing on a DCT intervention not in use nationally, and three studies of DCT interventions intended for use exclusively by contact tracers or government public health analysts, a total of 18 studies were included in the review (Fig. 1). Additionally, a comprehensive search of grey literature and other information sources identified 128 potentially relevant records on DCT implementation, of which 41 were included in the review (Fig. 1).

### Characteristics of individual sources of evidence

The characteristics of the 18 included studies are presented in Table 1. These were conducted in Australia (n = 5,^5^^,^^14^, ^15^, ^16^, ^17^), Fiji (n = 1,^18^), Japan (n = 4,^19^, ^20^, ^21^, ^22^), New Zealand (n = 3,^23^, ^24^, ^25^), and Singapore (n = 5,^26^, ^27^, ^28^, ^29^, ^30^). Of these five countries, New Zealand was the only country to deploy a QR code location check-in DCT app at initial deployment, with the other four countries initially deploying Bluetooth-based proximity tracing DCT apps. Most (n = 12, 67%) studies were designed as cross-sectional studies and recruited participants via online surveys, with study populations including patients and visitors attending healthcare facilities, healthcare workers, research panel database members, employees, adult members of the public, and confirmed COVID-19 cases and their contacts listed in a public health data registry. The number of participants in these studies ranged from 18 to 27,036. Data collection occurred in 2020 for 13 studies, in early 2021 for two studies, and the date(s) of data collection was not stated in three studies.

**Table 1:** Characteristics of 18 included studies of adoption, use and effectiveness of DCT interventions in the Western Pacific Region.

Country/area	DCT characteristics 1. Name 2. Tool 3. Technology 4. Data processing	Study ID	Aim of study	Study design	Data collection period	Population description	Method of recruitment	Number of participants included in analysis
Australia	1. COVIDSafe 2. Smartphone application 3. Proximity trails (Bluetooth) 4. Centralised	Garrett 2021^14^	Assess attitudes towards three tracking technologies prior to the launch of COVIDSafe, and compare to usage of the COVIDSafe app after launch.	Repeated cross-sectional study	Apr-20	Representative sample of the Australian public aged 18 years and older stratified by gender, age and state.	Online survey	878
		Vogt 2022^5^	Assess the effectiveness and usefulness of COVIDSafe in New South Wales.	Mixed methods study	May-20 – Nov-20	All individuals with confirmed SARS-CoV-2 infection aged 12 years and older in NSW during the study period.	Public health data registry	619
		Thomas 2020^15^	Investigate the uptake of the Australian Government's COVIDSafe app among Australians and examine the reasons why some Australians have not downloaded the app.	Cross-sectional study	May-20	Australian residents aged 18 years or older. Participants were excluded if they were a health care professional or had been tested for COVID-19.	Online survey	1500
		Degeling 2021^16^	Report on six online deliberative workshops in New South Wales to provide recommendations on the appropriateness of using the COVIDSafe app to enhance contact tracing capacity in Australia.	Qualitative research	Jun-20 – Jul-20	Research volunteers and respondents to a social media campaign. Workshop participants were selected from the convenience sampling frame based on representative socio-demographic characteristics.	Voluntary	43
		Lockey 2021^17^	Profile adopters and non-adopters of Australia's COVIDSafe app.	Cross-sectional study	Jun-20 – Jul-20	Australian adults aged over 18 years	Research panel database	2575
Fiji	1. careFIJI 2. Smartphone application 3. Proximity trails (Bluetooth); QR code location check-in 4. Decentralised	Chand 2021^18^	Discuss rollout of the careFIJI app for COVID-19 contact tracing in Fiji.	Text and opinion	N/A	N/A	N/A	N/A
Japan	1. COCOA 2. Smartphone application 3. Proximity trails (Bluetooth) 4. Decentralised	Kawakami 2021^19^	Investigate the association of downloading a COVID-19 contact tracing app, the COVID-19 Contact Confirming Application (COCOA), released by the Japanese government, with worry about COVID-19 and psychological distress in a sample of employed adults in Japan.	Cohort study	May-20 – Aug-20	Full-time employees	Online survey	996
		Gotanda 2021^20^	Examine whether the practice of preventive measures against COVID-19 differs by one's level of trust in government.	Cross-sectional study	Aug-20 – Sep-20	Japanese individuals aged 15–79 years participating in the Japan COVID-19 and Society Internet Survey	Online survey	25,482
		Shoji 2021^21^	Examine the role of individual prosociality and other factors such as perceived risk and trust in government, in encouraging the usage of contact tracing apps in Japan.	Cross-sectional study	Dec-20	68,480 people were selected and invited to participate in the survey from 4.65 million registrants of a large survey company in Japan.	Online survey	5402
		Ishimaru 2021^22^	Identify industry and workplace characteristics associated with the downloading of COCOA, the COVID-19 contact tracing app in Japan.	Cross-sectional study	Dec-20	Participants in an online cohort study on COVID-19 and work.	Online survey	27,036
New Zealand	1. NZ COVID Tracer 2. Smartphone application 3. QR code location check-in 4. Decentralised	Tretiakov 2021^23^	Explore how users experience the NZ COVID Tracer app in their everyday contexts, and identify determinants of app use.	Qualitative research	Oct-20 – Nov-20	Residents in Auckland aged 18–64 years who were users of the NZ COVID Tracer app.	Online survey	34
		Gasteiger 2021^24^	Explore the barriers and facilitators to the general public's use of the NZ COVID Tracer app.	Cross-sectional study	Not stated	Adults 18 years and older participating in the COVID-19 Stress and Health study	Voluntary	380
		Ali 2022^25^	Describe usage behaviour, motivations for use and determinants of use of the NZ COVID Tracer app in the general population.	Cross-sectional study	Not stated	Self-selected respondents to online survey	Online survey	261
Singapore	1. TraceTogether 2. Smartphone application 3. Proximity trails (Bluetooth) 4. Centralised	Saw 2021^26^	Identify the characteristics of individuals or factors associated with voluntary downloads of TraceTogether in Singapore.	Cross-sectional study	Apr-20 – Jul-20	Adults aged 21 years or older who were resident in Singapore for a minimum of two years	Online survey	505
		Huang 2020^27^	Compare the performance of the contact tracing app "TraceTogether” with that of a wearable tag-based real-time locating system (RTLS).	Diagnostic test accuracy study	May-20	18 physicians during a 10-day posting at the National Centre for Infectious Diseases COVID-19 screening centre, and patients attending the centre over the same time period.	Employees	18
		Huang 2021^28^	Assess the awareness of, willingness to use (acceptance) and actual use (adoption) of TraceTogether in Singapore.	Cross-sectional study	Jul-20 – Dec-20	Patients and caregivers attending two outpatient clinics at Tan Tock Seng Hospital	Clinic patients	3240
		Huang 2022^29^	Assess the factors influencing the acceptance and adoption of TraceTogether during the COVID-19 pandemic.	Repeated cross-sectional study	Jul-20 – Feb-21	Patients and visitors of two outpatient clinics at Tan Tock Seng Hospital	Clinic patients	3943
		Lee 2021^30^	Examine normative influences (descriptive and injunctive norms) on TraceTogether device use for contact tracing purposes.	Repeated cross-sectional survey	Jan-21 – Feb-21	Representative sample of Singapore residents aged 21 years or older who had downloaded TraceTogether or received a token, sampled from a voluntary research panel.	Online survey	1137

### Synthesis of results

#### Implementation of digital contact tracing interventions

By March 2022, 17 of the 37 WPR countries and areas had implemented DCT interventions for COVID-19 (Table 2), with a date range for initial deployment from February 11 2020 (China, Republic of Korea) to November 19 2021 (Macao SAR). Of these 17 countries and areas, DCT interventions were introduced prior to the first reported COVID-19 case in Cook Islands (CookSafe launched on June 18 2020, first reported COVID-19 case on February 15 2022^11^), and during periods of low or zero reported COVID-19 cases in several countries. For example, in Brunei Darussalam the BruHealth app was launched on May 5 2020, by which time 141 cumulative cases had been reported to WHO, followed by a period of zero cases until August 3 2020.^11^ In Fiji, careFIJI was launched on June 21 2020, with 18 cumulative cases reported by the launch date, and several months of zero reported cases subsequently.^11^ DCT apps were also in use during periods of zero or very low reported COVID-19 incidence in Australia, China, Guam, Hong Kong SAR, Lao PDR, New Zealand and Viet Nam.^11^

**Table 2:** Overview of digital contact tracing (DCT) interventions in the Western Pacific Region.

Country/area^a^	DCT intervention	Date of initial deployment	DCT functions at initial deployment	DCT functions added after initial deployment	Adoption model (voluntary, mandatory)^c^	Information sources
Australia	COVIDSafe (smartphone app)	27/04/2020	BLE proximity tracing (OpenTrace), contact notification		Voluntary	Studies^5^^,^^14^, ^15^, ^16^, ^17^ Government website^31^^,^^32^
Brunei Darussalam	BruHealth (smartphone app)	14/05/2020	GPS geolocation tracking QR code location check-in, risk classification		Initially voluntary, later mandatory to leave home	Government website^33^ News media^34^
Cambodia	Stop COVID (smartphone app)	20/02/2021	QR code location check-in		Voluntary	News media^35^^,^^36^
China	Health code app (smartphone app)	29/02/2020	GPS geolocation tracking, centralized data aggregation/triangulation, self-assessment, risk classification	QR code location check-in	Mandatory	Studies^37^^,^^38^ News media^39^^,^^40^
Cook Islands	CookSafe and CookSafe+ (smartphone app, token)	18/06/2020	CookSafe: BLE proximity tracing (GAEN^b^), exposure notification	CookSafe+: QR code location check-in	Voluntary	News media^41^
Fiji	careFIJI (smartphone app, token)	21/06/2020	BLE proximity tracing (OpenTrace), contact notification	QR code location check-in	Voluntary	Study^18^ News media^42^^,^^43^
Guam	COVID Alert (smartphone app)	10/09/2020	BLE proximity tracing (GAEN), exposure notification		Voluntary	Government website^44^ News media^45^^,^^46^
Hong Kong SAR (China)	LeaveHomeSafe (smartphone app)	16/11/2020	QR code location check-in		Initially voluntary, later mandatory in some settings	Government website^47^^,^^48^ News media^49^
Japan	COCOA (smartphone app)	19/06/2020	BLE proximity tracing (GAEN), exposure notification		Voluntary	Studies^19^, ^20^, ^21^, ^22^ Government website^50^
Lao PDR	Lao KYC (smartphone app)	29/04/2020	GPS geolocation tracking	QR code location check-in	Voluntary	News media^51^^,^^52^
Macao SAR (China)	Health Code (smartphone app)	19/11/2021	QR code location check-in		Initially voluntary, later mandatory in many settings	News media^53^^,^^54^
Malaysia	MySejahtera, MyTrace (smartphone app)	16/04/2020	MySejahtera: QR code location check-in	MyTrace: BLE proximity tracing (Google/Apple/Huawei)	Voluntary	Government website^55^ News media^56^^,^^57^
New Zealand	NZ COVID Tracer (smartphone app)	20/05/2020	QR code location check-in, exposure notification	BLE proximity tracing (GAEN)	Voluntary	Studies^23^, ^24^, ^25^ Government website^58^
Philippines	StaySafe (national government endorsed app), along with Traze (government transport sector only) and RC143 (non-government sector app), which are required to be integrated into StaySafe (smartphone apps).	03/09/2020	BLE proximity tracing (GAEN), GPS geolocation tracking, QR code location check-in, exposure notification	GPS geolocation tracking feature removed. Traze integrated with StaySafe.	Initially voluntary, later mandatory in some settings	Government website^59^, ^60^, ^61^ News media^62^
Republic of Korea	Corona map, Corona 100m, Now and here, Cobaek, (smartphone apps)	11/02/2020	GPS geolocation tracking, exposure notification, contact notification, centralized data aggregation/triangulation	QR code location check-in	Voluntary	Studies^63^^,^^64^
Singapore	TraceTogether (smartphone app, token)	20/03/2020	BLE proximity tracing (BlueTrace), exposure notification, contact notification	SafeEntry: QR code location check-in, contact notification	Initially voluntary, later mandatory in many settings	Studies^26^, ^27^, ^28^, ^29^, ^30^ Government website^65^^,^^66^ News media^67^
Viet Nam	Bluezone (smartphone app)	18/04/2020	BLE proximity tracing (GAEN), exposure notification, publication of case information		Initially voluntary, later mandatory in many settings	Studies^68^ Government website^69^ News media^70^^,^^71^

a No DCT interventions were implemented in American Samoa, French Polynesia (France), Kiribati, Marshall Islands, Micronesia, Federated States of, Mongolia, Nauru, New Caledonia (France), Niue, Northern Mariana Islands, Commonwealth of the (USA), Palau, Papua New Guinea, Pitcairn Island (UK), Samoa, Solomon Islands, Tokelau (New Zealand), Tonga, Tuvalu, Vanuatu, and Wallis and Futuna (France).

b GAEN; Google/Apple Exposure Notification.

c Adoption model refers to whether the DCT intervention was deployed on a voluntary or mandatory basis in the general population. It does not refer to model of adoption for specific populations, such as international travellers, who may be subject to mandatory use of DCT interventions even if use is voluntary in the general population.

At initial deployment, eight WPR countries and areas deployed Bluetooth low energy (BLE)-based proximity tracing apps, of which three countries (Australia, Fiji, Singapore) used the OpenTrace protocol and five used the Google/Apple Exposure Notification (GAEN) protocol (Cook Islands, Guam, Japan, Philippines, and Viet Nam) (Table 2). Three countries initially implemented DCT interventions based on QR code location check-in technology (Cambodia, Malaysia, and New Zealand). GPS tracking was used in Brunei Darussalam, China, Lao PDR, Philippines, and Republic of Korea.

Several countries modified their DCT interventions over time (Table 2). Singapore introduced a physical token for BLE-based proximity tracing for users without access to smartphones. BLE-based proximity tracing was added to the DCT apps in Malaysia and New Zealand. QR code location check-in functionality was ultimately implemented in 13 of the 17 countries/areas with DCT interventions. China integrated QR code location check-in as part of its informatics approach to retrieve and update an individual's “health code” that determines access to public spaces, as well as quarantine, isolation, and testing requirements.^37^^,^^38^ NZ COVID Tracer in New Zealand was the first DCT app to support manual in-app location check-in as an alternative to QR code check-in. The Philippines mandated the integration of several sector-specific and non-government DCT apps into the StaySafe app.^59^ Ten countries and areas deployed DCT interventions as voluntary interventions, whereas seven countries and areas mandated their use, including six that launched DCT as a voluntary tool but later mandated use in some or most public settings (Table 2). From late 2021 to early 2022, DCT apps have been withdrawn from use or scaled back due to changes in COVID-19 control strategies following vaccine rollout and substantially-increased incidence^11^ due to highly transmissible Omicron variants. In Fiji, as of February 2022, the careFIJI app is no longer required for entry into businesses and venues, as location-based contact tracing is not currently part of its COVID-19 response.^42^ In Hong Kong SAR, in light of the surge in case numbers and demand for testing, LeaveHomeSafe also stopped alerting users about premises visited by COVID-19 cases to conserve testing resources for those at higher risk of infection or most vulnerable to COVID-19.^47^ In Australia, the definition of close contact was narrowed, with a focus on household or household-like contacts, as part of the response to the Omicron wave.^31^ In Singapore, health authorities initially planned to continue the use of TraceTogether until COVID-19 is no longer epidemic,^67^ but requirements for use of the TraceTogether app were substantially reduced in April 2022.^65^

#### Adoption and use

Adoption and use measures were reported for 13 of 18 published studies (Table 3). Ten studies reported the proportion of DCT app downloads (adoption) amongst study participants,^14^, ^15^, ^16^, ^17^^,^^19^^,^^21^^,^^22^^,^^25^^,^^26^^,^^28^ six studies reported on active or regular DCT use,^5^^,^^14^^,^^20^^,^^24^^,^^25^^,^^29^ and one study reported time to adoption of DCT app.^14^ Nine studies reported adoption or use outcomes in different study population subgroups.^5^^,^^17^^,^^19^, ^20^, ^21^, ^22^^,^^25^^,^^26^^,^^28^ Estimates of adoption and use reflect different study populations, time periods, implementation phases, and COVID-19 epidemiological contexts. In Australia, estimates of downloads of the COVIDSafe app in the adult population ranged from 33%^16^ to 47%,^14^ whereas estimates of active use ranged from 26.8% to 38.3% in a representative sample of the adult population,^14^ and 22% amongst confirmed adult COVID-19 cases.^5^ In Japan, the percentage of study populations who had downloaded COCOA ranged from 14.6%^21^ to 25.1%,^22^ whereas estimates of national adoption ranged from 17.6%^21^ to 20.8%^22^ at the time the studies were conducted.

**Table 3:** Adoption and use of digital contact tracing (DCT) interventions in the Western Pacific Region from 18 published studies.

Country	DCT name	Study ID	Adoption/use measures reported	Description of adoption/use measures reported	Factors associated with adoption/use (positive, negative or both)	Description of barriers and enablers of adoption/use reported
Australia	COVIDSafe	Garrett 2021^14^	Downloads; Regular users; Time to adoption (not reported separately for different populations)	An estimated 44% of the sampled population downloaded the app. Most downloaded within the first day (29%) or week (57%) after launch. Active use amongst those who had downloaded COVIDSafe (defined as installation, registration and Bluetooth switched on when in public) was 61%–87% in the sampled population over the duration of the study. Inferring from study participants' reports of the percentage of family and friends who have downloaded the app, it was estimated that 47% of the Australian population would download and use COVIDSafe. Actual download rate was slower than predicted rate based on earlier surveys about hypothetical tracking technologies.	Trust in authorities (+) Perceived benefit to community (+) Privacy concerns and beliefs (−) Perceived benefit to individual (−) Technical features/issues (−) Perceived effectiveness (−) Peer group effects (+)	App users cited compliance with government policy, concern for others' health, concern for own health, and desire for return to normal activities. Non-users cited factors including lack of trust in public figures and science, privacy concerns, battery usage, and a belief the app will not be effective. Future intent to download COVIDSafe related to technological issues, time, not leaving the house, and 'waiting on others'.
		Vogt 2022^5^	Active use by cases (reported separately for different populations)	137 (22%) of COVID-19 cases used the app during their infectious period. Cases who used the app were less likely to live in socioeconomically disadvantaged areas, were more likely to be born in Australia, and acquired infection from a contact outside their household.	Technical features/issues (−)	Barriers to use were reported for public health staff to access COVIDSafe data. which included technical issues (under- or over-detection of contacts on different types of phones, and app not recording a single contact during the case's infectious period).
		Thomas 2020^15^	Downloads (not reported separately for different populations)	37.3% downloaded COVIDSafe, 18.7% intended to, 27.7% refused, and 16.3% were undecided.	Privacy concerns and beliefs (−) Access to digital devices/internet (−) Trust in authorities (−) Perceived effectiveness (−) Technical features/issues (−) Peer group effects (−)	Privacy and data security concerns were the main barrier to adoption, reported by 25% of those who refused or were undecided about adopting COVIDSafe. 24.1% cited technical issues, such as phones being too old or limitations in data consumption and storage space. Other reasons included belief that social distancing was sufficient and the app was unnecessary, distrust in government, questioning app's effectiveness, wanting more information, and peer influence.
		Degeling 2021^16^	Downloads (not reported separately for different populations)	By late September 2020, it was estimated that 7 million people had registered their details via the app, representing approximately 20% of the entire population or approximately one third (7/18 million) of all smartphone owners.	Privacy concerns and beliefs (+/−) Trust in authorities (+/−) Benefit to community (+) Technical features/issues (−) Perceived effectiveness (−)	Voluntariness was central to the app's acceptability, for security and privacy reasons as well as concerns about inequalities in smartphone access. Majority of participants considered the privacy and personal protections enacted in the COVIDSafe app design and supporting legislation as appropriate. The minority who believed legislation went too far had low trust in government competence to maintain data security, and overall low trust in the government COVID-19 response. A smaller minority felt that the legislation did not go far enough given the scale of the threat to lives and health. Privacy and security concerns did not vary with current or possible future COVID-19 incidence. Uncertainty about app effectiveness was repeatedly raised as a concern. Other factors included cyber security competency, Bluetooth security, and introduction of mandated use in some contexts despite national legislation against mandates. No factors were explicitly linked to actual usage.
		Lockey 2021^17^	Downloads (reported separately for different populations)	Download frequency assessed across seven profiles comprising indicators of education, age, income, dispositional desire for privacy and political ideology. Download frequency ranged from 32.8% to 59.8% across profiles, with an average download frequency of 43% in the sampled population.	Privacy concerns and beliefs (+/−) Trust in authorities (+) Demographic and socioeconomic characteristics (+)	Factors associated with downloading COVIDSafe included being more educated, more conservative, wealthier, a lower dispositional desire for privacy, and higher trust in government. Dispositional desire for privacy associated with lower app download.
Fiji	careFIJI	Chand 2021^18^	N/A	None	Access to digital devices/internet (+/−) Privacy concerns and beliefs (−) Perceived benefit to individual (+)	Barriers cited include incompatible mobile devices, insufficient digital literacy, QR scanner failures due to unstable internet connection, general privacy concerns, increased battery consumption especially on lower-end devices, and literacy levels. Enablers cited include role of telecommunications providers (Vodafone Fiji Ltd) in reducing price of smartphones, reimbursing data (100 MB upon download of app, 10 times more than required for app download). Another possible enabler is that app users can avoid queuing to sign in manually to locations, making it easier to maintain social distance.
Japan	COCOA	Kawakami 2021^19^	Downloads (reported separately for different populations)	20.4% downloaded app, who were more likely to be male, older, living with a child, university graduates, or working from home	None reported	None reported
		Gotanda 2021^20^	Use (not further defined) (reported separately for different populations)	Unadjusted estimates for use of a contact tracing app are not provided. Amongst individuals with “high trust in government”, use was estimated at 20.3%. Amongst individuals with “low trust in government”, use was estimated at 14.6%.	Trust in authorities (+/−)	High trust in government was associated with 20.4% use of a contact tracing app vs 14.6% use amongst respondents with low trust in government.
		Shoji 2021^21^	Downloads (reported separately for different populations)	Nationally, the adoption frequency was 17.6% on December 28 2020. In the study population, 14.6% adopted COCOA.	Trust in authorities (+) Community attachment (+) Perceived benefit to individual (+) Demographic and socioeconomic characteristics (+)	Males, university graduates and those with regular jobs were more likely to use COCOA. Agreeableness, attachment to the community, concern about health risks, concern about social risks and trust in national government were associated with app adoption. These factors varied by age.
		Ishimaru 2021^22^	Downloads (reported separately for different populations)	In the study population, 25.1% reported having downloaded COCOA (compared to 20.8% download frequency in general population at 18 March 2021). Participants in the public service and information technology sector were more likely to download the app than participants in other industries. Participants in large companies were more likely to use the app than participants in small companies. Risk perception, health behaviour, and demographic characteristics had limited effect on adoption.	None reported	None reported
New Zealand	NZ COVID Tracer	Tretiakov 2021^23^	N/A	N/A; study population restricted to app users	Trust in authorities (+) Community attachment (+) Perceived benefit to individual (+) Population-level COVID-19 risk (+/−)	Supporting contact tracing in the context of controlling the pandemic was the strongest perceived benefit. Individual health benefits including option to self-isolate early after an exposure event were also described, but less frequently and often only after specific prompting. Reduction in uncertainty about exposure and infection risk was also cited as a benefit. Privacy was a concern for some participants, but amongst the app-using study population, did not deter adoption and use. Participants expressed high levels of trust in the New Zealand government, and app use was seen as a civic duty. Active use varied with the COVID outbreak context, with use declining at low alert levels.
		Gasteiger 2021^24^	Regular users (not reported separately for different populations)	55% of respondents were using the app frequently or sometimes, and 45% had not used it.	Privacy concerns and beliefs (−) Trust in authorities (+) Perceived effectiveness (+) Support to businesses (−) Technical features/issues (−) Population-level COVID-19 risk (+/−)	Changing perception of COVID-19 risk according to local outbreak context. Lack of business support also cited as a barrier. Government communications and recommendations facilitated use, as did perceived importance of app for contact tracing. A minority reported privacy concerns, including fear of hackers and misuse of data to record movements of users. Government mass surveillance was also a concern.
		Ali 2022^25^	Downloads; Regular users (reported separately for different populations)	92.7% respondents had downloaded NZ COVID Tracer ‘at some point’. 38.7% used 'all the time', 32.6% used 'most of the time'.	Community attachment (+) Privacy concerns and beliefs (+) Access to digital devices/internet (+) Trust in authorities (+) Perceived benefit to family or peer group (+) Perceived benefit to individual (+) Demographic and socioeconomic characteristics (+)	79.3% respondents used NZ COVID Tracer to show responsibility to community, 68.6% to protect family and friends, 75.9% to help stop the outbreak, 51% to know the risk of infection, 44% for peace of mind, 42.5% to help stay healthy, 34.1% to reduce mortality in older people. 28.4% respondents strongly agreed that the NZ COVID Tracer app provider would protect personal data, 38.3% agreed, 28.7% were neutral. Statistically significant predictors of app use were age, smartphone ownership/use, and trust in data privacy protection. Several technical and design features were noted, but not explicitly analysed in terms of association with app use. Lack of function to record check-out time was the main design feature disliked about the app (36.8%). Issues such as lack of Bluetooth or GPS functionality, bugs and errors, battery consumption, slow app speed, and app use taking too long were reported by <20% respondents. 31.4% reported no issues with the app. 87% reported that the app is easy to use.
Singapore	TraceTogether	Saw 2021^26^	Downloads (reported separately for different populations)	54.3% had downloaded TraceTogether.	Trust in authorities (+) Adoption of other health behaviours (+) Demographic and socioeconomic characteristics (not assoc.) Population-level COVID-19 risk (not assoc.)	The number of behavioural modifications made in response to the COVID-19 pandemic (e.g. hand sanitising, mask wearing) and confidence in the government was associated with adoption of Trace Together. Demographic characteristics, local COVID-19 cases, and lockdown status were not associated with adoption.
		Huang 2020^27^	N/A	Not applicable	None reported	None reported
		Huang 2021^28^	Downloads/receipt of token (reported separately for different populations)	49% adopted the app or token overall. Adoption frequency increased over the six month study duration, reaching 70% in younger adults and 79% in older adults amongst smartphone users. Amongst older adults without a smartphone, adoption increased from 8% to 47% following distribution of tokens.	Access to digital devices/internet (−)	Adoption frequency was lower for non-smartphone users.
		Huang 2022^29^	Current active users at time of survey (not reported separately for different populations)	56.8% overall use, rising from 38.4% use in Jul–Oct 2020 to 85.1% use by Jan–Feb 2021	Privacy concerns and beliefs (−) Population-level COVID-19 risk (−) Perceived benefit to community (+) Peer group effects (+)	Respondents who perceived TraceTogether as useful and necessary had higher likelihood of acceptance. Concerns about personal data collected by TraceTogether was associated with lowered willingness to accept the app. Peer effects motivated app use, low perceived population-level COVID-19 risk is associated with lower app use. Older adults, employed respondents, and tertiary educated respondents were more likely to adopt TraceTogether.
		Lee 2021^30^	N/A	Study was restricted to population that had downloaded app or received token. 46.3% reported using TraceTogether always in the last seven days. 22.8% used TraceTogether for more than six months, 16.4% had used for less than one month.	Community attachment (+) Peer group effects (+) Demographic and socioeconomic characteristics (+/−)	Descriptive and injunctive norms, stronger community perception were positively associated with intention to use TraceTogether. Intention to use TraceTogether also varied amongst ethnic groups.

One study in New Zealand with self-selected respondents to an online survey reported that 92.7% of respondents had downloaded the NZ COVID Tracer app, and 71.3% reported using the app either ‘all the time’ or ‘most of the time’.^25^ Another study reported that an estimated 55% of those who had downloaded NZ COVID Tracer were regular users.^24^ In Singapore, estimates for adoption (including downloads of TraceTogether app or receipt of token) ranged from 49%^28^ to 54.3%^26^ overall, though reached 79% in some subpopulations.^28^ Estimates of active or regular use were similar, around 56.8% overall, reaching 85.1% in some subpopulations.^29^ No quantitative estimates of adoption or use were provided for Fiji in the included study.

For countries and areas with no data on adoption or use from the 18 published studies, there was limited information on adoption and use in the grey literature. In Fiji, news media reported that less than 10% of the population had downloaded careFIJI by September 2020.^43^ In Guam, approximately 28% of the population had downloaded the COVID-19 Alert app by November 2020.^45^ As of February 2021, the LeaveHomeSafe app in Hong Kong SAR had been downloaded 840,000 times since launch,^49^ equivalent to approximately 13% of the adult population. As of December 2020, the MySejahtera app in Malaysia had approximately 24.5 million users, approximately 70% of the total population.^56^ As of October 2020, approximately 4% of the population of the Philippines had downloaded StaySafe.^62^ In Viet Nam, more than 22.5 million downloads of the Bluezone app were recorded by December 2020,^70^ approximately one-third of the adult population.

Determinants of adoption and use across the 18 studies are presented in Table 3 and summarised in Table 4. A wide range of potential determinants of adoption and use were investigated, including level of trust in public authorities, privacy beliefs and concerns, perceived benefits of DCT use to individuals, perceived benefits of DCT interventions for communities, perceived effectiveness of DCT interventions, and others. Overall, trust in authorities, and privacy concerns and beliefs were the most frequently identified determinants of adoption and use, though with mixed findings. In nine studies,^14^^,^^16^^,^^17^^,^^20^^,^^21^^,^^23^, ^24^, ^25^, ^26^ trust in authorities was associated positively with DCT use, whereas in three studies,^15^^,^^16^^,^^20^ distrust in authorities was associated with refusal or delay in adopting or using DCT interventions. Conversely, privacy concerns and beliefs were negatively associated with adoption or use in seven studies,^14^, ^15^, ^16^, ^17^, ^18^^,^^24^^,^^29^ and positively associated with use in three studies.^16^^,^^17^^,^^25^ Perception of low effectiveness of DCT interventions was more often cited as a reason for not using DCT apps (three studies^14^, ^15^, ^16^), rather than positive perceptions of the effectiveness of DCT apps motivating use (one study^24^). Five studies^5^^,^^14^, ^15^, ^16^^,^^24^ reported that technical features and issues with DCT apps were a barrier to adoption and use, of which four were in Australia. Access to digital devices and the internet was identified as an enabler of use in two studies,^18^^,^^25^ and a barrier to use in three studies.^15^^,^^18^^,^^28^ Most studies did not systematically identify which factors were investigated but not associated with adoption or use, making systematic analysis across studies vulnerable to publication bias and missing data.

**Table 4:** Summary of determinants of adoption or use of digital contact tracing (DCT) interventions from 15 of 18 published studies reporting data on barriers and enablers of adoption or use.

Determinant of adoption or use	Positively associated with adoption or use (n studies)	Negatively associated with adoption or use (n studies)	Not associated with adoption or use (n studies)
Trust in authorities	9	3	N/A^a^
Benefit to community	3	0	N/A
Benefit to individual	4	1	N/A
Privacy concerns and beliefs	3	7	N/A
Technical features/issues with the DCT intervention	0	5	N/A
Perceived effectiveness of the DCT intervention	1	3	N/A
Access to digital devices/internet	2	3	N/A
Perceived population-level COVID-19 risk or alert level	2	3	1
Community attachment	4	0	N/A
Peer group effects	3	1	N/A
Demographic and socioeconomic characteristics	4	1	1

a N/A indicates that there was no data available to evaluate whether a determinant of adoption of use was included in a study and found not to be associated with adoption or use, or whether the determinant was not investigated in a study.

#### Effectiveness

Two studies reported the sensitivity, specificity, and positive and/or negative predictive values of DCT interventions. One study in Singapore compared TraceTogether to a wearable real-time locating system (RTLS) tag amongst 18 physicians coming into contact with hospital staff, patients and visitors over a 10-day period in May 2020.^27^ When validated against electronic medical records, TraceTogether had a sensitivity of 0.0%, specificity of 98.4%, positive predictive value of 0.0%, and negative predictive value of 79.2%. RTLS had a much higher sensitivity (96.9%), lower specificity (83.1%), and higher positive (59.6%) and negative (99.0%) predictive values. Nevertheless, wearable RTLS was considered impractical for community-wide implementation.^27^

The second study in Australia compared the COVIDSafe app to conventional contact tracing for all adult COVID-19 cases in the state of New South Wales from May to November 2020.^5^ This study found that COVIDSafe had low sensitivity (15%) and positive predictive value (39%) for identifying close contacts, and only 17 unique close contacts were identified through COVIDSafe that were missed through conventional contact tracing, which represented 0.07% of close contacts recorded during the study period. Additionally, COVIDSafe use was lower amongst COVID-19 cases (22%) than the general population (44%, as reported in^14^). The low sensitivity of COVIDSafe led to additional time spent by contact tracers to classify app-suggested contacts, which delayed contact notification in some instances.^5^

Low adoption and use, along with under-performance of DCT apps on iPhones compared to Android phones were reported in both studies as contributing to the low sensitivity of proximity tracing interventions.^5^^,^^27^

## Discussion

### Summary of evidence

DCT interventions were implemented in 17 WPR countries and areas to support the COVID-19 response, with notable variation in design and implementation characteristics. The WPR represents a region of particular interest regarding implementation of DCT interventions, as the WPR was where COVID-19 was first detected, countries and areas in the WPR were amongst the earliest adopters of DCT technologies and a wide range of DCT technologies were used, including BLE proximity tracing (with both the GAEN and OpenTrace protocols in use), QR code location check-in apps, GPS geolocation tracking, and centralised data aggregation. It is also the only region where DCT interventions were introduced in countries and areas prior to their first confirmed COVID-19 case. Use of DCT interventions was mandated in several countries but was voluntary in most countries. Most of the 20 countries and areas that did not deploy DCT interventions were Pacific Island countries and territories, which reflects the absence of community-acquired COVID-19 cases in many Pacific Island countries until late 2021 or early 2022,^11^ as well as variable population-level access to a smartphone to support DCT implementation.^72^ However, it is notable that several Pacific Island countries and areas deployed DCT interventions during periods of zero or very limited local transmission, in preparation for initiating an outbreak response, including the Cook Islands, Fiji, and Guam.

There was limited consistency in the measures of adoption, use or effectiveness reported by studies. Most studies estimated adoption or use at a single time point, often shortly after app launch, and frequently in non-representative populations (e.g., research volunteers, respondents to online surveys advertised through social media, and patients and visitors in hospital settings). Factors associated with DCT intervention adoption and use in the WPR were similar to other studies, including a global systematic review that identified privacy concerns, trust, and perceived benefit as being most frequently associated with adoption.^73^ No study aimed to investigate DCT adoption or use in remote, disadvantaged, or vulnerable populations, and inequalities in access and use were not addressed in most studies, despite being an important determinant of effective population coverage of DCT interventions.^13^ Only two studies^14^^,^^25^ reported active or regular use as well as adoption, both of which reported that regular use amongst adopters ranged from 61% to 87%. These differences may be attributable to low COVID-19 case numbers at the time the studies were conducted, technical issues with DCT apps, and the health intention-behaviour gap that affects use of many health interventions.^14^^,^^25^ This suggests that for the majority of studies that reported adoption rather than use, adoption substantially overestimates actual use. The distinction between adoption and use is particularly relevant for DCT interventions that require regular active participation of users, such as QR code check-in apps, though even more passive technologies such as proximity tracing apps still require that a smartphone is switched on and with Bluetooth enabled after initial download (adoption). Only one study^5^ from Australia investigated active use of a DCT intervention amongst COVID-19 cases and compared this to estimated use in the general population, which is a recommended indicator for evaluation of proximity tracing DCT apps.^13^ It is also notable that most studies were based on data collected in 2020, with no study reporting on data collected after February 2021.

Similarly to an earlier global review of the public health effectiveness of DCT interventions,^4^ evidence of effectiveness of DCT interventions in the WPR is lacking overall. Since the prior review, two additional studies have been published that reported effectiveness measures for DCT interventions in the WPR. Of these, only the study in New South Wales, Australia comprised an effectiveness evaluation in the general population, whereas the study in Singapore compared the TraceTogether app effectiveness to a wearable technology in a small pilot study comprising 18 physicians and their contacts in a hospital outpatient setting (a COVID-19 testing clinic). Both studies were conducted in low transmission settings compared to later time periods, though the impact of the transmission context on measures of effectiveness is unclear. Both COVIDSafe and TraceTogether were based on a centralised model for contact identification and notification, which may not have been scalable to higher transmission settings even if the sensitivity of these tools for detecting contacts was higher. The effectiveness evaluation in New South Wales was also the only study that investigated the impact of DCT interventions on the contact tracing workforce,^5^ which showed adverse impact without a clear public health benefit. In other settings in the WPR, it is unclear how DCT interventions complemented or were integrated into conventional contact tracing workflows. No study evaluated the effectiveness of QR code location check-in apps, despite this being the most commonly used technology. The limited evidence of effectiveness of DCT interventions in the WPR is consistent with limited evidence for DCT interventions in general, including for COVID-19^4^ and other outbreak-prone infectious diseases where DCT interventions have been tested, such as Ebola and pertussis.^74^

### Recommendations for further research and practice

Digital contact tracing interventions are expected to have highest utility when case incidence exceeds conventional contact tracing capacity,^1^^,^^3^ but in the WPR, there is no published research relating to adoption, use or effectiveness of DCT interventions since the emergence of more transmissible variants. Given that many WPR countries and areas did not experience sustained community transmission until 2021 or later, and DCT interventions were introduced or remained in use during these periods, this represents an important evidence gap, as evidence on adoption, use and effectiveness of DCT interventions from the first year of the COVID-19 pandemic may not be generalisable to time periods dominated by more transmissible variants. Furthermore, most WPR countries and areas have significantly scaled back contact tracing in 2022, coinciding with the highest incidence rates reported so far in the pandemic, and achievement of high COVID-19 vaccine coverage.^11^ These national responses across the WPR are broadly inconsistent with WHO advice to prioritise rather than abandon contact tracing efforts in the context of high transmission.^75^ The guidance recommends continued contact tracing for contacts at highest risk of infection and/or severe COVID-19 disease, and carefully calibrating the contact definition and quarantine duration to reduce transmission whilst minimising adverse societal and other impacts.^75^ However, as there is very limited evidence for the effectiveness of DCT interventions in the WPR, and indeed globally, it is difficult to make recommendations for ongoing use of DCT interventions for COVID-19 or other infectious diseases. The available evidence in the WPR is limited to two effectiveness evaluations of BLE-based proximity tracing apps, the findings of which suggest that BLE-based proximity tracing technologies may have limited effectiveness due to low adoption, use, and effectiveness at detecting contacts.

Given the scale of national investment in and encouragement of adoption and use of DCT interventions throughout the WPR, the most significant recommendation arising from this review is to strengthen the evidence base for the public health effectiveness of DCT interventions. No evaluations have been published of QR code location check-in apps, and no specific framework for their evaluation has been developed, despite being the most common technology for DCT in the WPR. There are important conceptual differences between QR code location check-in apps and proximity tracing apps that would merit the development of specific indicators. For example, adoption is likely to be much less informative for QR code location check-in apps, given that users must actively “check-in” for contact tracing data to be generated. The development of more expansive evaluation frameworks for a wider range of DCT technologies, as well as post-deployment evaluation studies and other research are required to understand the relative contribution of different types of DCT interventions to reducing COVID-19 exposure and transmission events. However, the privacy-preserving protocols of many DCT smartphone apps specify that user data is deleted after a specified amount of time, and for some apps, contact tracing data was not centrally stored (i.e. decentralised), therefore DCT data may no longer be available for analysis. Future DCT interventions should be designed to enable real-time or retrospective data analysis and evaluation using deidentified data, balanced against privacy concerns, and aim to report indicators of effectiveness that can ascertain public health effectiveness overall.^13^

Considering challenges related to adoption, use and effectiveness of DCT interventions, conventional contact tracing is still likely to be required in many contexts, including for COVID-19 and other infectious diseases where contact tracing can reduce transmission. Using conventional and digital contact tracing approaches concurrently could make up for the gaps and limitations of each approach alone. Notwithstanding the observed limitations of the current generation of DCT interventions, DCT continues to have potential to minimise recall bias and identify missed contacts, allowing faster contact notification and quarantine, and enabling systems to scale up faster and with fewer resources than a manual approach, especially in settings with high population adoption and use.

### Limitations

There were several important limitations to this review. Overall, most limitations related to the operational nature of this research. For example, only one reviewer conducted the final selection, data extraction, and analysis of peer-reviewed literature for the adoption, use and effectiveness outcomes, which may have led to relevant studies being missed, or other types of bias. Due to the very wide range of types of information sources retrieved through the systematic and open-ended search, including published studies, government websites and media releases, online news media, and other information sources, the quality and consistency of each individual source of information was not separately appraised. Though multiple reviewers were involved in identifying grey information sources, this occurred as a sequential process over the course of the operational response to the COVID-19 pandemic. Therefore, discrepancies between reviewers were not identified. This review does not specifically address DCT design features and functionalities that support or enable the public health outcomes of adoption, use and effectiveness. For example, the Mobile App Rating Scale (MARS) has been widely used to assess the quality of mHealth apps, with assessment domains including functionality, aesthetics, and in-app information.^76^ As the relationship between app design features and public health outcomes of DCT apps has not been established in the literature, these additional data items were not collected in this review. Though automatic online translation services were used for screening and analysis of non-English language information sources, some sources of information published in languages other than English may have been missed.

### Conclusions

There is limited high-quality evidence available to evaluate the contribution of DCT interventions to mitigating COVID-19 transmission in the WPR. In particular, very little evidence is available on DCT adoption, use, or effectiveness during transmission waves attributed to highly transmissible variants of concerns, when high case incidence means that conventional contact tracing is not feasible. An important element of future pandemic preparedness is to continue to research and improve DCT interventions, including addressing technical issues and improving privacy features to facilitate adoption and use. Development or application of robust evaluation frameworks^13^ for evaluation of DCT interventions prior to any future implementation, including ensuring the need for data availability for research and evaluation is balanced against privacy preserving protocols, is imperative to avoid replicating the DCT effectiveness evidence gap observed during the COVID-19 response to date. Additionally, a strong community engagement strategy to build trust and boost DCT adoption and use, as part of an effort to increase trust in public health authorities more broadly, should be an integral part of preparedness planning for health emergencies.

## Contributors

GG conceptualised the research and guided the research project. MBT, MC, VC and AM conducted the literature search, collected and analysed data, and contributed to the data interpretation. MBT wrote the original draft of the manuscript. MBT, MC, VC, AM and GG revised and edited the manuscript.

## Data sharing statement

As this study was a systematic scoping review of published studies and grey literature sources, all data are publicly available in the specified references.

## Declaration of interests

The authors declare no competing interests.

## References

[1] Ferretti L., Wymant C., Kendall M. (2020). Quantifying SARS-CoV-2 transmission suggests epidemic control with digital contact tracing. Science.

[2] Whitelaw S., Mamas M.A., Topol E., Van Spall H.G.C. (2020). Applications of digital technology in COVID-19 pandemic planning and response. Lancet Digit Health.

[3] Abueg M., Hinch R., Wu N. (2021). Modeling the effect of exposure notification and non-pharmaceutical interventions on COVID-19 transmission in Washington state. Npj Digit Med.

[4] Mazza C., Girardi D., Gentile L., Gaeta M., Signorelli C., Odone A. (2021). Public health effectiveness of digital contact tracing in the COVID-19 pandemic: a systematic review of available data. Acta Bio-Medica Atenei Parm.

[5] Vogt F., Haire B., Selvey L., Katelaris A.L., Kaldor J. (2022). Effectiveness evaluation of digital contact tracing for COVID-19 in New South Wales, Australia. Lancet Public Health.

[6] Poletto C., Boëlle P.-Y. (2022). Learning from the initial deployment of digital contact tracing apps. Lancet Public Health.

[7] Wymant C., Ferretti L., Tsallis D. (2021). The epidemiological impact of the NHS COVID-19 app. Nature.

[8] World Health Organization (2021). Weekly epidemiological update on COVID-19 - 28 December 2021. https://www.who.int/publications/m/item/weekly-epidemiological-update-on-covid-19---28-december-2021.

[9] Patel J., Sridhar D. (2020). We should learn from the Asia–Pacific responses to COVID-19. Lancet Reg Health – West Pac.

[10] Summers J., Cheng H.-Y., Lin H.-H. (2020). Potential lessons from the Taiwan and New Zealand health responses to the COVID-19 pandemic. Lancet Reg Health - West Pac.

[11] (2022). WHO coronavirus (COVID-19) dashboard. https://covid19.who.int.

[12] Tricco A.C., Lillie E., Zarin W. (2018). PRISMA extension for scoping reviews (PRISMA-ScR): checklist and explanation. Ann Intern Med.

[13] European Centre for Disease Prevention and Control, World Health Organization (2021). https://data.europa.eu/doi/10.2900/642768.

[14] Garrett P.M., White J.P., Lewandowsky S. (2021). The acceptability and uptake of smartphone tracking for COVID-19 in Australia. PLoS One.

[15] Thomas R., Michaleff Z.A., Greenwood H., Abukmail E., Glasziou P. (2020). Concerns and misconceptions about the Australian government's COVIDSafe app: cross-sectional survey study. JMIR Public Health Surveill.

[16] Degeling C., Hall J., Johnson J., Abbas R., Bag S., Gilbert G.L. (2021). Should digital contact tracing technologies be used to control COVID-19? Perspectives from an Australian public deliberation. Health Care Anal HCA J Health Philos Policy.

[17] Lockey S., Edwards M.R., Hornsey M.J., Gillespie N., Akhlaghpour S., Colville S. (2021). Profiling adopters (and non-adopters) of a contact tracing mobile application: insights from Australia. Int J Med Inf.

[18] Chand S.S., Chand A.A., Chand K.K. (2021). The use of careFiji app for contact tracing during the COVID-19 pandemic: digital gap and challenges faced in Fiji. Int J Surg.

[19] Kawakami N., Sasaki N., Kuroda R., Tsuno K., Imamura K. (2021). The effects of downloading a government-issued COVID-19 contact tracing app on psychological distress during the pandemic among employed adults: prospective study. JMIR Ment Health.

[20] Gotanda H., Miyawaki A., Tabuchi T., Tsugawa Y. (2021). Association between trust in government and practice of preventive measures during the COVID-19 pandemic in Japan. J Gen Intern Med.

[21] Shoji M., Ito A., Cato S. (2021). Prosociality and the uptake of COVID-19 contact tracing apps: survey analysis of intergenerational differences in Japan. JMIR MHealth UHealth.

[22] Ishimaru T., Ibayashi K., Nagata M. (2021). Industry and workplace characteristics associated with the downloading of a COVID-19 contact tracing app in Japan: a nation-wide cross-sectional study. Environ Health Prev Med.

[23] Tretiakov A., Hunter I. (2021). User experiences of the NZ COVID tracer app in New Zealand: thematic analysis of interviews. JMIR MHealth UHealth.

[24] Gasteiger N., Gasteiger C., Vedhara K., Broadbent E. (2021). The more the merrier! Barriers and facilitators to the general public's use of a COVID-19 contact tracing app in New Zealand. Inf Health Soc Care.

[25] Ali Z.S., Dang H. (2022). Factors impacting the use of the NZ COVID Tracer application in New Zealand. Smart Health.

[26] Saw Y.E., Tan E.Y.-Q., Liu J.S., Liu J.C.J. (2021). Predicting public uptake of digital contact tracing during the COVID-19 pandemic: results from a nationwide survey in Singapore. J Med Internet Res.

[27] Huang Z., Guo H., Lee Y.-M., Ho E.C., Ang H., Chow A. (2020). Performance of digital contact tracing tools for COVID-19 response in Singapore: cross-sectional study. JMIR MHealth UHealth.

[28] Huang Z., Guo H., Lim H.Y., Chow A. (2021). Awareness, acceptance, and adoption of the national digital contact tracing tool post COVID-19 lockdown among visitors to a public hospital in Singapore. Clin Microbiol Infect.

[29] Huang Z., Guo H., Lim H.Y.-F., Chow A. (2022). Determinants of the acceptance and adoption of a digital contact tracing tool during the COVID-19 pandemic in Singapore. Epidemiol Infect.

[30] Lee J.K., Lin L., Kang H. (2021). The influence of normative perceptions on the uptake of the COVID-19 TraceTogether digital contact tracing system: cross-sectional study. JMIR Public Health Surveill.

[31] Australian Government Department of Health (2021). AHPPC statement on testing, tracing, isolating and quarantining in high levels of COVID-19 community transmission. Aust. Gov. Dep. Health.

[32] Health AGD of (2020). COVIDSafe: New app to slow the spread of coronavirus. Aust. Gov. Dep. Health.

[33] Ministry of Health - bruhealth (2022). https://www.moh.gov.bn/SitePages/bruhealth.aspx.

[34] Brunei (2021). Govt announces that only people with BruHealth app allowed to go out. The Star. https://www.thestar.com.my/aseanplus/aseanplus-news/2021/09/25/brunei-govt-announced-that-only-people-with-bruhealth-app-allowed-to-go-out.

[35] Bunthoeurn O. (2022). Cambodia's ‘Stop Covid’ QR Code wins international award. https://www.phnompenhpost.com/national/cambodias-stop-covid-qr-code-wins-international-award.

[36] Kongnov Tith (2021). https://www.khmertimeskh.com/50816331/stop-covid-qr-code-takes-off/.

[37] Fan Y., Wang Z., Deng S., Lv H., Wang F. (2022). The function and quality of individual epidemic prevention and control apps during the COVID-19 pandemic: a systematic review of Chinese apps. Int J Med Inf.

[38] Ye Q., Zhou J., Wu H. (2020). Using information technology to manage the COVID-19 pandemic: development of a technical framework based on practical experience in China. JMIR Med Inform.

[39] Business NG and DC CNN (2021). https://www.cnn.com/2020/04/15/asia/china-coronavirus-qr-code-intl-hnk/index.html.

[40] (2022). Health QR codes in full effect in Hangzhou.

[41] (2021). Covid-19 tracing app available for download.

[42] Kate T. (2022). https://www.fijitimes.com/carefiji-app-and-qr-not-required-for-entry-anymore/.

[43] (2020). Fijians encouraged to download COVID-19 contact tracing app after slow uptake.

[44] (2022). Privacy policy | Guam Covid Alert. https://guamcovidalert.guam.gov/resources/privacy-policy/.

[45] Wen A. (2021). https://www.guampdn.com/story/news/local/2020/11/02/covid-19-alert-app-downloaded-guam-residents-contact-tracing/6111248002/.

[46] Pacific News Center (2020). https://www.pncguam.com/guam-launches-covid-alert-mobile-app-to-aid-contact-tracing-efforts/.

[47] Shuk-kwan C. (2022). https://www.thestandard.com.hk/breaking-news/section/4/187048/LeaveHomeSafe's-notification-is-not-a-legally-binding-testing-request:-Chuang-Shuk-kwan.

[48] (2022). Launch of ‘LeaveHomeSafe’ COVID-19 exposure notification mobile app. https://www.fehd.gov.hk/english/licensing/guide_general_reference/COVID19_LeaveHomeSafe.html.

[49] Reuters (2021). Hong Kong sees rush for burner phones as government pushes contact-tracing app. https://www.reuters.com/world/china/hong-kong-sees-rush-burner-phones-government-pushes-contact-tracing-app-2021-02-18/.

[50] 新型コロナウイルス接触確認アプリ（COCOA) (2022). https://www.mhlw.go.jp/stf/seisakunitsuite/bunya/cocoa_00138.html.

[51] Phonevilay L. (2021). https://laotiantimes.com/2021/07/05/laos-covid-taskforce-unveils-new-contact-tracing-qr-code/.

[52] Lao P.D.R. (2020). MPT announces Coronavirus tracking mobile app and website. DataGuidance.

[53] (2021). Macao Health Code app with contact tracing function ready for download.

[54] (2022). Health Code contact tracing to become ‘mandatory’ at eateries by CNY.

[55] (2022). MySejahtera. https://mysejahtera.malaysia.gov.my/intro_en/.

[56] MOK O (2020). https://www.malaymail.com/news/malaysia/2020/12/04/health-ministry-source-mysejahtera-covers-24.5-million-users-with-up-to-300/1928682.

[57] (2022). MySejahtera replaces check-out feature with Bluetooth based ‘MySJ Trace’. The Star.

[58] (2022). NZ COVID tracer app. NZ COVID tracer app. https://tracing.covid19.govt.nz.

[59] Aguilar K. (2021). Palace: use of StaySafe app to be fully implemented in 10 days. https://newsinfo.inquirer.net/1406149/palace-use-of-staysafe-app-to-be-fully-implemented-in-10-days.

[60] Stay Safe — Stay Healthy (2022). Stay safe. http://staysafe.ph/.

[61] Presidential Communications Operations Office (2020). https://pcoo.gov.ph/news_releases/ph-government-launches-staysafe-ph-as-official-contact-tracing-program-palace/.

[62] (2021). DOTr turns back on gov’t-endorsed StaySafe.

[63] Lee C.S. (2021). Contact tracing apps for self-quarantine in South Korea: rethinking datafication and dataveillance in the COVID-19 age. Online Inf Rev.

[64] Park S., Choi G.J., Ko H. (2020). Information technology–based tracing strategy in response to COVID-19 in South Korea—privacy controversies. JAMA.

[65] Ministry of Health, Singapore (2022). Further easing of community and border measures. https://www.moh.gov.sg/news-highlights/details/further-easing-of-community-and-border-measures.

[66] (2022). TraceTogether. https://www.tracetogether.gov.sg/.

[67] Puthucheary J. (2022). https://www.channelnewsasia.com/singapore/covid19-tracetogether-safeentry-moh-parliament-safe-management-measures-2534451.

[68] Tran T.P.T., Le T.H., Nguyen T.N.P., Hoang V.M. (2020). Rapid response to the COVID-19 pandemic: Vietnam government's experience and preliminary success. J Glob Health.

[69] MOH Viet Nam, MIC Viet Nam (2020). https://bluezone.ai/Bluezone_White_paper_en.pdf.

[70] (2021). Bluezone becomes top-downloaded app again.

[71] (2022). NCOVI and Bluezone in Vietnam.

[72] Mccool J., Hill J., Dobson R., Whittaker R. (2020). Access to ICT in the pacific Islands region: a brief report: ICT in the pacific Islands. Pac Health Dialog.

[73] Oyibo K., Sahu K.S., Oetomo A., Morita P.P. (2022). Factors influencing the adoption of contact tracing applications: systematic review and recommendations. Front Digit Health.

[74] Anglemyer A., Moore T.H.M., Parker L. (2020). Digital contact tracing technologies in epidemics: a rapid review. Cochrane Database Syst Rev.

[75] World Health Organization (2022). https://apps.who.int/iris/rest/bitstreams/1411031/retrieve.

[76] Stoyanov S.R., Hides L., Kavanagh D.J., Zelenko O., Tjondronegoro D., Mani M. (2015). Mobile app rating scale: a new tool for assessing the quality of health mobile apps. JMIR MHealth UHealth.

