# Supplementary material for: Systematic scoping review of the implementation, adoption, use, and effectiveness of digital contact tracing interventions for COVID-19 in the Western Pacific Region
Source: Lancet Reg Health West Pac. 2023 Feb 25;34:100647. doi: 10.1016/j.lanwpc.2022.100647 (PMC9958511; doi:10.1016/j.lanwpc.2022.100647)
Supplement: Supplementary File S2 [file mmc2.docx]

Caption for Supplementary File

Preferred Reporting Items for Systematic Reviews and Meta-Analyses (PRISMA) extension for Scoping Reviews checklist
